# Closed Cell Rigid Polyurethane Foams Based on Low Functionality Polyols: Research of Dimensional Stability and Standardised Performance Properties

**DOI:** 10.3390/ma13061438

**Published:** 2020-03-21

**Authors:** Agnė Kairytė, Arūnas Kremensas, Giedrius Balčiūnas, Sylwia Członka, Anna Strąkowska

**Affiliations:** 1 Laboratory of Thermal Insulating Materials and Acoustics, Institute of Building Materials, Faculty of Civil Engineering, Vilnius Gediminas Technical University, Linkmenu st. 28, LT-08217 Vilnius, Lithuaniagiedrius.balciunas@vgtu.lt (G.B.); 2 Institute of Polymer and Dye Technology, Lodz University of Technology, Zeromskiego 116, 90-924 Lodz, Poland; sylwia.czlonka@edu.p.lodz.pl (S.C.); anna.strakowska@p.lodz.pl (A.S.)

**Keywords:** polyurethane foam, waste-based polyol, dimensional stability, performance properties, circular economy

## Abstract

Currently, polyurethane foam producers come across the several problems when petroleum-based polyols are replaced with low functionality biomass, or waste-based, polyols. In addition, the dilemma is intensified with regulations that require full or partial replacement of blowing agents that can cause high ozone depletion with alternatives like water, which causes the formation of CO_2_. Therefore, these gases diffuse out of the foam so quickly that the polymeric cell walls cannot withstand the pressure, consequently causing huge dimensional changes at ambient temperature and humidity. Even though the theoretical stoichiometric balance is correct, the reality shows that it is not enough. Therefore, polyethylene terephthalate waste-based polyol was chosen as a low functionality polyol which was modified with high functionality sucrose-based polyol in order to obtain dimensionally stable polyurethane foams in the density range of 30–40 kg/m^3^. These more stable foams are characterized by linear changes no higher than 0.5%, short-term water absorption by partial immersion no higher than 0.35 kg/m^2^, and water vapor resistance factors up to 50. In order to obtain thermally efficient polyurethane foams, conventional blowing agents and water systems were implemented, thus, assuring thermal conductivity values in the range of 0.0198–0.0204 W/(m·K) and obtaining products which conform to all the requirements for performance of sprayed and factory-made polyurethane foam standards EN 14315-1 and EN 13165.

## 1. Introduction

The desire to either maintain current or improve future living conditions has forced the public to pay attention to ecological issues in all areas of life, especially in construction. Currently, the main objective of the construction industry is to contribute to the development of eco-friendly materials through the use of environmentally friendly technical and design solutions that ensure energy conservation through the use of local renewable materials or wastes with low cost, appealing aesthetics, and minimal environmental impact.

The quality of the interior environment of a building is a major health factor, given that people spend most of their time indoors. In this respect, green building materials make an important contribution to a healthy environment. Manufacturers of building materials must contribute, not only to raw materials, resource management, and waste minimization, but also to the production of materials with adequate physical-mechanical properties [1]. The intensified bio-economy forces the polyurethane industry to develop either partially or completely ecological polyurethane foam (PUR) from renewable sources, such as liquefied soybean stalks [2], corn stalks [3,4], wheat stalks [5,6,7], paper waste [8], bark and starch [9,10], avocado seeds [11], and lignin [12,13]. However, synthetic materials, specifically plastics, are the most widely used in the construction industry because of their durability and low weight.

The rapidly growing amount of plastic waste causes a major negative impact on living organisms. Since 2015, 6.3 billion metric tons of plastic waste have been accumulated, but only 9% of it has been recycled, while 79% was landfilled and 12% was incinerated [14], thus, causing extreme environmental pollution. Polyethylene teraphthalate (PET) is the most commonly used plastic, which finds its application in repositories for liquids and foods due to its low price, transparency, and sufficient mechanical performance. The approximate amount of PET products consumed exceeds 24 million per year, and the amount proceeds to grow [15], contributing a significant volume of plastic waste. Consequently, new technologies to utilize PET wastes are in high demand to maximize recycling and avoid landfilling or incineration. The problem of PET waste could be solved by physical or chemical utilization [16,17]. One of the major advantages of chemical utilization is the production of aromatic polyester polyols (APPs), which are used to form polyurethane foams. The use of renewable resources in the synthesis of chemicals reduces the negative impact on the environment, arising from the use of scarce resources and the release of greenhouse gases. These resources open up the possibility of either partially or completely replacing polymers from petroleum products, which may compete with, or even surpass, conventional materials in terms of price, quality, and environmental impact. Due to its relatively low cost, diethylene glycol (DEG) is commonly used for the synthesis of APPs from PET waste. However, the DEG/PET waste system product has some drawbacks. First, the final product is a viscous liquid, which solidifies at room temperature. Secondly, the transesterification product is not compatible with industrial blowing agents, so adipic acid and glycerol are also used for APP production [18]. Whereas all the studies made on PET waste-based polyols are dedicated to the synthesis of adhesives and coatings [19,20,21], there are few studies which analyze polyurethane foams for thermal insulation purpose but there is no data on problems which are met by the producers of closed cell rigid polyurethane foams—the obtained PET waste-based APPs, when used in the water-blown polyurethane synthesis, cause dimensional instabilities, which are not favored in testing laboratories and, most importantly, in construction sites. Additionally, authors [22,23,24] analyze mechanical properties and thermal degradation of the obtained PET waste-based polyurethane foams, however, apart compressive stress/strength and thermal stability it is of great importance to assure initial dimensional stability and prevent low functionality polyols-based foams from shrinking or post-blowing. Therefore, it is necessary to evaluate if the polyurethane foams conform the requirements of European Normative in order to be implemented and supplied into the market.

Therefore, the purpose of this study is to modify unstable factory-made and sprayed rigid PET waste-based polyurethane foams and to yield foams with improved dimensional stabilities with no more than a 5% change in length and width and 10% change in thickness and densities of 30 to 40 kg/m^3^. More importantly, the basic performance properties for thermal insulating foams, such as thermal conductivity, water absorption, water vapor permeability, thermal stability and flammability, are tested, and the microstructure is analyzed.

## 2. Experimental

### 2.1. Case Study 

The use of APPs in polyurethane foam systems is limited only by low functionality (≤ 2), which is determined by the glycol used in the production of APPs. Polyurethane foam made from such polyols exhibits dimensional changes exceeding standard values. (Reported standards are ≤ 5% variation in length and width and ≤ 10% in thickness [25] or ≤ 15% variation in length and width and ≤ 10% thickness [26]). Therefore, multifunctional, low molecular weight polyols can be used to modify these properties.

**Figure 1:**
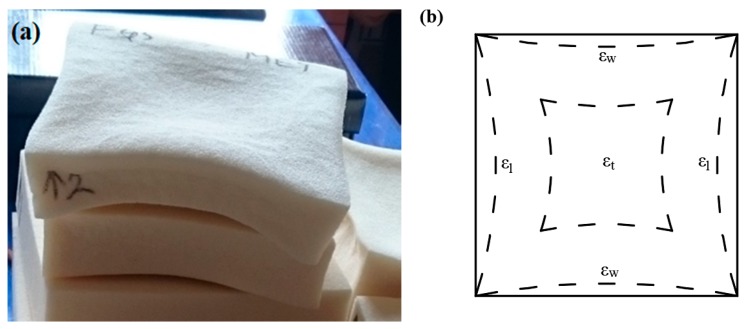
Polyurethane foam specimens: (**a**) after preparation for thermal conductivity tests and (**b**) graphical interpretation of dimensional changes.

Currently, several manufacturers come across the above-mentioned problems when petroleum-based polyols are replaced with low functionality biomass or waste-based polyols. In addition, the dilemma is intensified with regulation to either fully or partially replace blowing agents that cause high ozone depletion with alternatives, e.g., water, which is basically the cause of CO_2_ formation. Therefore, these gases diffuse out of the foam so quickly that the polymeric cell walls cannot withstand the pressure, consequently, causing huge dimensional changes at ambient temperature and humidity conditions, as shown in Figure 1.

In this study, focus is placed on the formulations, which, according to the molar ratios of components A and B, are correctly calculated without taking the PET-based polyol’s functionality into account (Table 1).

The above presented formulations need to be improved in order to get a product with a density between 30 and 40 kg/m^3^ (based on a customer’s order), dimensional stability at both ambient and increased temperatures, and humidity conditions which conform to the requirements for factory-made [25] and sprayed [26] polyurethane foams. In order to achieve this aim, additional measures should be considered.

### 2.2. Raw Materials

For the study of dimensionally stable, rigid polyurethane foam, the polyester polyol NEOPOLYOL 240 (JSC Neo Group, Klaipėda, Lithuania), made from recycled PET waste, and the polyol PETOL PZ 400-4G (Oltchim, Râmnicu Vâlcea, Romania), were used. The most important chemical characteristics are given in Table 2.

For the formation of the rigid polyurethane foam structure, a blowing agent system was selected from water, which emits CO_2_ gas during its reaction with isocyanate, and fluorinated hydrocarbon Solkane 365 mfc (Solvay, Riga, Latvia). The amount of water used in the production of the rigid polyurethane rigid foams ranged from 2 to 3.5 parts by weight (pbw), and Solkane 365 mfc ranged from 15 to 29 pbw. A polyether modified dimethyl-polysiloxane surfactant, Struksilon 8006 (Brenntag, Kędzierzyn-Koźle, Poland), was used to form the polyurethane foam. Based on the manufacturer’s recommendations, 2.0 pbw of the surfactant were used to reduce surface tensions and to form and stabilize the porous structure. Polycat 9 (Air Products and Chemical, Inc., Decatur, AL, USA) was used as a catalyst for controlling the main reaction time for the rigid polyurethane foam.

The flame retardant ROFLAM P (PCC Rokita SA, Brzeg Dolny, Poland) was used to ensure the flammability characteristics of the final products. 4,4’-diphenylmethane diisocyanate (referred to as isocyanate) from Lupranat M20S (BASF, Ludwigshafen, Germany) with an average functionality of 2.7 and a reactive group (-NCO) content of 31.5% was used to cure the rigid polyurethane foam. All formulations used an isocyanate index of 100.

### 2.3. Preparation of PET Waste-Based Polyurethane Foams

For the polyurethane foam formation, the polyols, catalyst, blowing agents, flame retardant, and surfactant were agitated for 10 min in a blowing device tank at 1800 rpm (component A). The isocyanate in the component B tank was sprayed in a 1:1 ratio with component A. The amount of isocyanate required to react with each of the -OH groups in the polyols and water was calculated by Equations (1)–(3):

(1) $$E_{MDI}=\frac{4200}{\%NCO}$$

(2) $$E_{P}=\frac{56,100}{n_{OH}}$$

(3) $$m_{MDI}=\left(\frac{I_{MDI}}{100}\right)\cdot E_{MDI}\cdot \left(\frac{m_{P}}{E_{P}}+\frac{m_{H_{2}O}}{E_{H_{2}O}}\right)$$

where *E_MDI_* is the isocyanate equivalent weight (g/mol), and %*NCO* is the percentage of isocyanate reactive groups (%). The value of 56,100 describes the molecular weight of KOH (mg/mol), and 4200 is the molecular weight of isocyanate (mg/mol). *E_P_* is the equivalent weight of the polyol system (g/mol), *n_OH_* describes the hydroxyl value of the polyol system (mg/KOH g), *m_MDI_* is the isocyanate amount (pbw), and *I_MDI_* is the isocyanate index (d.m.). *m_P_* describes the amount of the polyol system (pbw), *m_H2O_* the amount of water (pbw), and *E_H2O_* the equivalent weight of water (g/mol). To evaluate the stability and performance of the rigid polyurethane foam with an apparent density between 30 and 40 kg/m^3^, the compositions shown in Table 3 were investigated.

Each of the resulting blends was sprayed from an industrial polyurethane foam spraying device. The mixtures were blended for 10 s and sprayed on a 500 × 500 × 100 mm^3^ plywood sheet, on which they were left, blown freely, at 23 ± 5 °C. Before the tests, specimens were conditioned, at least 24 h before testing but for a maximum of 8 days, in an environment with a temperature of 23 ± 5 °C and a relative humidity of 50 ± 5%.

### 2.4. Test Methods Used for the Mixtures and Polyurethane Foam

Foaming characteristics of polyurethane foaming mixture were determined in accordance with [26] by a cup test method with a digital thermometer TS-131 with a probe having an accuracy of 0.5 °C and a digital timer. The length and width of the specimens were determined in accordance with [27] and the density according to standards from [28]. The thermal conductivity before and after aging the specimens for 21 days was determined in accordance with the requirements of [25,26,29] (Annex C) with one specimen symmetric configuration horizontal flow meter FOX 304 with active specimen edges protection in the bottom-up direction of the heat flow. The measuring range of the device is from 0.01 to 0.50 W/(m·K), with an accuracy of 1%. The thermal conductivity of the specimens 300 × 300 × 50 mm^3^ was determined at an average temperature of 10 °C, and the difference between the lower and upper panels was 20 °C during the tests. Ten specimens for each composition were tested to ensure the results were reliable.

In order to determine the effect of different blowing agents and polyol systems on the moisture properties of the products, the short-term water absorption of the products after partial immersion for 24 h was determined for specimens with a 200 × 200 × 50 mm^3^ volume, according to method B [30]. Specimens with a size of 100 × 100 × 50 mm^3^ were used for water vapor permeability tests, in accordance with standard [31]. The climatic conditions of the test were as follows 23-0/50: Δ*p* was 1400 Pa, the average air temperature was 22.8 °C, and the average air pressure was 745 mmHg. The specific air permeability (*δ_air_*) during the test was 0.717. The test assemblies were filled with calcium chloride and were weighted at regular intervals of no less than 24 h. The direction of the water vapor flow relative to the product surface was perpendicular to the surface of the test object.

Shrinkage was evaluated for 300 × 300 × 50 mm^3^-sized specimens, which were cut for thermal conductivity test 2 days after production and measured both immediately and 1 h after cutting the length, width, and thickness directions. The shrinkage was calculated according to the following equation:

(4) $$\Delta \varepsilon =\frac{b_{1}-b_{2}}{b_{1}}\times 100$$

where Δ*ε* is the shrinkage (%), *b_1_* is the dimension of the sample cut 2 days after production (mm), and *b_2_* is the respective dimension of the sample 1 h after cutting (mm).

To evaluate the durability of the products, a dimensional stability test was carried out according to the methodology provided by [32]. Specimens were tested at 70 ± 2 °C and 90% ± 5% relative air humidity for 48 h using a climatic chamber (Feutron 3522/51) with a temperature measurement range of 30–100 °C with an accuracy of 0.2 °C and a humidity measurement range of 10–100% with an accuracy of 5%. Three 200 × 200 × 50 mm^3^ specimens were tested for each composition. For the evaluation of the structural parameters, the percentage of closed cells was determined according to Method 2 from source [33] for three specimens of each composition with dimensions of 100 × 30 × 30 mm^3^.

Thermogravimetry (TGA) and differential thermogravimetry (DTG) were conducted under the air atmosphere using STA 449 F1 Jupiter Analyzer (Netzsch Group, Erlangen, Germany) at temperature interval from 25 to 600 °C. Temperature raising speed was 10 °C/min.

The limited oxygen index (LOI) was obtained using an Oxygen Index Instrument (NETZSCH TAURUS Co., Ltd., Weimar, Deutschland). The size of the samples was (120 × 10 × 10) mm^3^. A sample tip was ignited for 5 s by means of a gas burner supplied with a propane-butane mixture. The limited oxygen index was calculated as the percentage of oxygen and nitrogen volume in the mixture.

## 3. Results and Discussion

### 3.1. Characteristics of the Mixtures and Apparent Density of the Foams

The reaction kinetics of the polyurethane foam mixtures mainly depend on the rate of blowing and of the gelling reactions. On the other hand, the properties of the products themselves depend on the polyol functional groups and the number of hydroxyls [34]. These and other characteristics of the mixtures, where a part of the traditional polyol is replaced by PET waste-based polyol, are shown in Table 4.

It may be observed that PUR compositions presented by the manufacturers (the control) have lower functionality compared to those with adjusted compositions. The foaming process is determined by measuring the characteristic processing times, cream, string-gel and tack-free times and sometimes reaction temperatures. Therefore, the obtained results are presented in Table 5.

**Figure 2:**
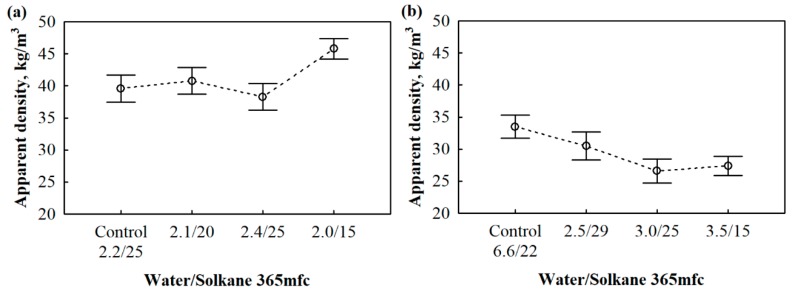
Effect of different rigid polyurethane foam compositions on apparent density variation: (**a**) 40 kg/m^3^ and (**b**) 30 kg/m^3.^

As the results show, all compositions are characterized by a high reactivity and temperature which are basically determined by a high amount of blowing catalysts, i.e., 4.2 pbw. However, a slight difference occurred in control systems where glycerol is involved as a co-polyol. As it has been already determined in [35] that glycerol acts as a start time extender. Taking into consideration the scattering of the results, the impact of blowing agent ratio is negligible because the rate of a reaction is assured through the balance of blowing and gelling catalysts. Furthermore, the control composition with a density of 40 kg/m^3^ has a higher hydroxyl value because of the glycerol used, which has increased the consumption of isocyanate in the system. Assumedly, glycerol was used to stabilize the PUR sample by increasing the content of hard segments. Apparently, the amount of cross linker is low, and further addition is not economical. As a previous study showed [36], it is necessary to replace 10% to 15% of low-functionality polyol with glycerol to obtain dimensionally and structurally stable polyurethane foams. The apparent density of the polyurethane foam is one of the key parameters in determining the properties of the finished product, so it is important to evaluate the effect that each composition has on this parameter. Since the main goal is to develop a stable, rigid polyurethane foam with an apparent density of 30 to 40 kg/m^3^, the average values for this characteristic are given in Figure 2.

The main raw materials that determine the density change are the blowing agents, which, in this case, are water and Solkane 365 mfc. Table 2 shows that the compositions use different water/Solkane 365 mfc ratios for these blowing agents, i.e., 2.1/20, 2.4/25, 2.0/15, 2.5/29, 3.0/25, and 3.5/18. A review of the literature has shown that the apparent density of the product decreases with increasing blowing agent content [37,38], but with 30 kg/m^3^ foams, the apparent density decreases with decreasing content of the Solkane 365 mfc in water/Solkane 365 mfc compositions of 3.0/25 and 3.5/18. A similar trend is observed in foams with an apparent density of 40 kg/m^3^. The difference between the results is explained by the use of water in the polyurethane foam. Water is characterized by a better blowing efficiency compared to Solkane 365 mfc.

Thus, compared to control polyurethane foams with densities of 30 kg/m^3^, polyurethane foams have 9.0%, 21%, and 18% lower apparent densities at water contents of 2.5, 3.0, and 3.5 pbw, respectively. Meanwhile, compared to the control foam with a density of 40 kg/m^3^, the parameter was reduced by 3.3% in the 2.4/25 water/Solkane 365 mfc system. This reduction in the apparent density can be assigned to the amount of isocyanate introduced to the polyol system. The same observation was made during studies of petrochemical polyol (OH = 449 mg KOH/g) replacement with bio-polyol (OH = 276 mg KOH/g) [34], which showed that the reduced hydroxyl value of the polyol system reduced the amount of isocyanate needed.

### 3.2. Dimensional Stability of the Products

Due to the rapid expansion of the rigid polyurethane foam during the foaming process, the molecular chains were quickly stretched, causing internal stress and crystallinity that was too low to be stereotyped at room temperature. The CO_2_ concentration in the cells was higher than in the atmosphere, causing the CO_2_ inside the foam to diffuse out and shrink the foam [39]. As rapid diffusion of the blowing agent through the cell walls destroys polymer stability [40], the product standard [25,26] does not specify requirements for dimensional changes after production and specimen cutting. Therefore, there are no requirements for dimensional stability at ambient temperature for sprayed PUR foams, either. 

**Figure 3:**
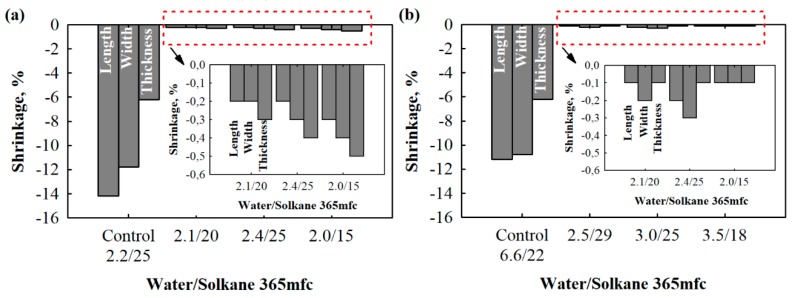
Shrinkage of specimens cut for thermal conductivity test 2 days after production: (**a**) 40 kg/m^3^ and (**b**) 30 kg/m^3^.

There is little information in the literature on dimensional changes of polyurethane foams, but many researchers modify existing polyols to increase the number of functional groups in order to avoid excessive shrinkage and negative linear changes in temperature and humidity conditions [34,41]. According to the experience of other scientists, a traditional polyether polyol having a functionality of 4.3 has been used for dimensional stabilization. As can be seen from Table 2, glycerol was eliminated from the adjusted compositions. Additionally, the amount of functionality was increased with the addition of a sucrose-based polyol. The results from dimensional stability tests (Figure 3) show that the adjusted compositions are characterised by a stabilised structure and dimensions after production. However, as can be observed from Figure 3, control compositions for PUR with densities of 40 kg/m^3^ (2.2/25 water/Solkane 365 mfc) and 30 kg/m^3^ (6.6/22 water/Solkane 365 mfc) are distinguished as improper for the use of testing and, especially, application in building envelopes. This was because the average changes for 40 kg/m^3^ PUR foam were 14.2% and 11.8% for length and width and 6.2% for thickness, and deviations of 11.2% and 10.8% for length and width and 6.2% for thickness were observed for 30 kg/m^3^ PUR foam.

**Figure 4:**
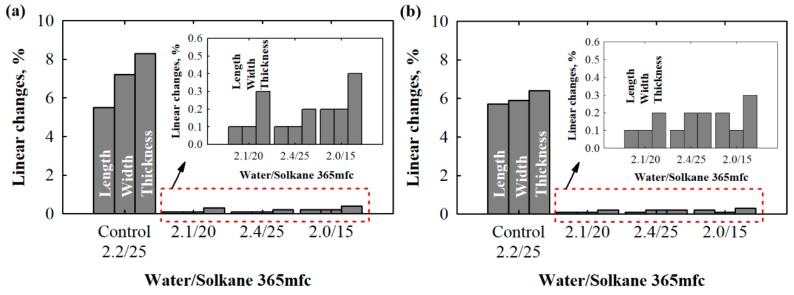
Linear changes after treatment at 70 °C and 90% relative humidity: (**a**) 40 kg/m^3^ and (**b**) 30 kg/m^3^.

Contrary results are presented for the PUR foams with adjusted compositions (Figure 4). Both 30 and 40 kg/m^3^ products have sufficient dimensional stability, as defined by the product standard. It can be observed that denser polyurethane foams tend to be more deformable.

Again, this can be explained by the fact that 40 kg/m^3^ polyurethane foam contains 10% more lower-functionality polyols from PET waste compared to 30 kg/m^3^ polyurethane foam, resulting in a lower number of functional groups. In any case, dimensional changes were reduced to a maximum of 0.5%. The obtained results are similar to those for polyurethane foams with filler- and liquid-based modifications and, in some cases, even better than those at 20 °C [42,43], indicating that the obtained foams could even be used in harsh conditions.

Although the possibilities of shrinkage at ambient temperature are not specified, the requirements for dimensional changes under increased temperature and humidity conditions are clearly indicated. Figure 4 presents the average percent values of dimensional changes after the maintenance of specimens at 70 °C and 90% humidity for 2 days.

In order to reduce gas diffusion through the walls of the foam cells into the environment and the negative effects of this diffusion, such as shrinkage of the rigid polyurethane foam at 23 °C and 50% relative air humidity and linear dimensional changes at higher temperatures and humidity, the polyol or polyol system should have more than 3 functional groups (Table 4).

### 3.3. Thermal Conductivity and Microstructure of the Stable Products

When rigid polyurethane foam is used for thermal insulation of building envelopes, it is important to evaluate the thermal insulating properties. Most values of thermal conductivity, i.e., ~65–80%, include thermal conductivity of a gas or gas mixture [44]. This basic property of thermo-insulating polyurethane foam also depends on the type of gas used in production and its diffusion rate from the product. This gas in the cells is replaced by air over a short period of time, so it is very important to evaluate the change in this parameter over time. Table 6 shows the variation of initial thermal conductivity, thermal conductivity after aging, and structural parameters depending on the product composition.

According to the product standards from [25,26], both sprayed and factory-made polyurethane foams have a mandatory shelf life of up to 8 days for measuring the initial thermal conductivity. It can be seen that the variation in average values of thermal conductivity for the 30 and 40 kg/m^3^ products before aging are very insignificant, i.e., the maximum possible difference between the lowest and highest values is 3.9% for 40 kg/m^3^ and 2.0% for 30 kg/m^3^ products. Because the value of thermal conductivity for polyurethane foams depends on the type of blowing agent and its amount in the system, these small differences in average thermal conductivity values, prior to aging, between different apparent density products are due to small differences in the water/Solkane 365 mfc ratios. In this case, the replacement of petroleum-based polyol with 20% and 30% PET waste-based polyol has no effect on the thermal insulating properties. 

**Figure 5:**
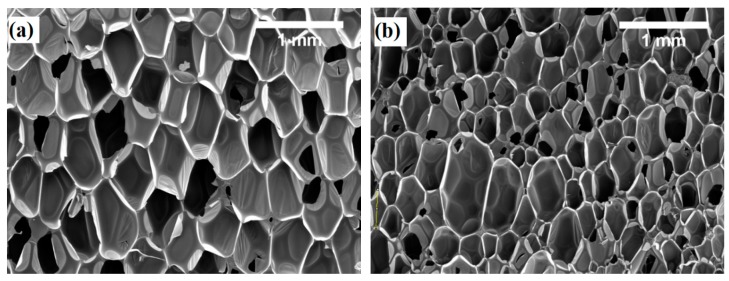
Microstructure of rigid polyurethane foam (magnification ×50) at an apparent density of (**a**) 30 kg/m^3^ and (**b**) 40 kg/m^3^.

In contrast, closed cell foams age over time; thus, an increase in their thermal conductivity can be observed (Table 6). This has been well-demonstrated for both polyurethane and polyisocyanurate foams with different blowing agents [45]. Therefore, the same trend can be observed when examining the thermal conductivity of products with an apparent density of 30 and 40 kg/m^3^ after aging for 21 days at 70 °C. When comparing the thermal conductivity values of all six compositions before and after aging, a significant difference can be observed. After the aging procedure, thermal conductivity increased by 27.6% at the water/Solkane 365 mfc ratio of 2.1/20. Similar variations were observed in other compositions. This can be explained by the fact that the composition uses water as an auxiliary blowing agent. During the isocyanate and water reaction, CO_2_ is generated, and it has a higher diffusion rate, while evaporation of Solkane 365 mfc from the product is a long process. Also, Table 4 shows that products with higher apparent densities exhibit cells that are almost 2 times larger (Figure 5). Others have observed that closed cells which are 2 times smaller determine lower thermal conductivity values [46], and based on the current results, the average thermal conductivity values for foams with a cell size of ~0.400 mm did not significantly differ from foams with an average cell size of ~0.800 mm. This difference can be attributed to the system of blowing agents because the aforementioned study discusses foams blown with only the CO_2_ generated during the isocyanate and water reaction.

In any given composition, the resulting products have an apparent density suitable for sprayed and factory-made rigid polyurethane foams ranging from 26.6 to 45.8 kg/m^3^ and lower thermal conductivity compared to other polymeric foams.

For example, expanded or extruded polystyrene foam and fibrous materials, such as glass or stone wool, range from 0.0251 to 0.0264 W/(m·K). The results obtained show that the value of the thermal conductivity is almost unchanged. However, other studies have obtained higher thermal conductivity values for PET waste-based polyurethane foams with similar apparent densities [41], but the difference may be explained by the implementation of different blowing agent systems and the nature of the cellular structure because the morphology of the polymer foams plays a crucial role in determining their thermal performance and aging [47].

### 3.4. Moisture Properties of the Stable Products

**Figure 6:**
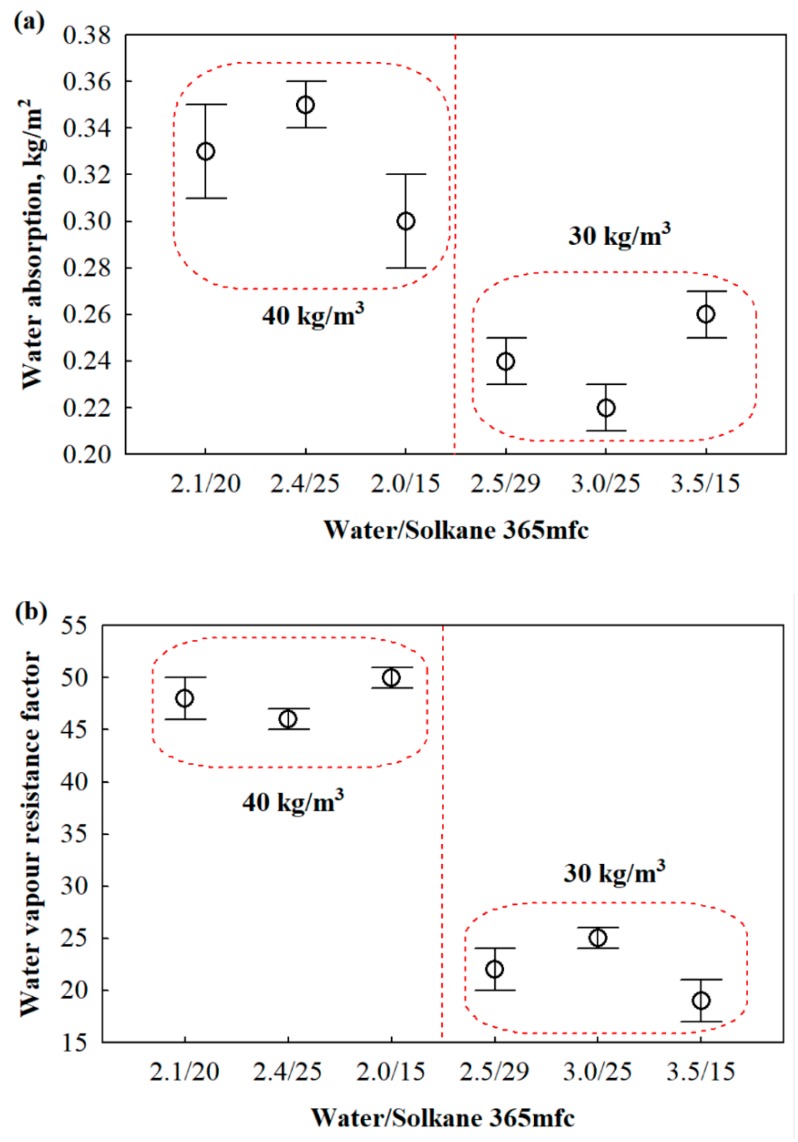
Moisture properties of rigid polyurethane foam: (**a**) short-term water absorption and (**b**) water vapor resistance factor.

Although there are no studies on the moisture properties of PET waste-based polyurethane foams, the use of thermal insulating materials in partitioning structures can result in water exposure (direct or vapor-type), and the intensity of this exposure affects the physical and mechanical properties of the thermal insulating layer. In this case, rigid polyurethane foam is used in well-insulated structures, which is why standards for both sprayed and factory-made polyurethane foams indicate short-term water absorption and vapor permeability tests (Figure 6).

From Figure 6a,b, it can be seen that as the percentage of closed cells increases (Table 6) and the product density decreases, the water vapor resistance factor and short-term water absorption also decrease. It can be concluded that water and water vapor more easily migrate in the structure with a relatively lower percentage of closed cells than at higher values of said parameter.

With an increase in the product density from 30 to 40 kg/m^3^, the average content of closed cells is reduced by 4.5%, while the short-term water absorption and water vapor resistance factor increase by 38% and 118%, respectively. Additionally, the difference in the percentage of closed cell contents may be the key factor for increased water absorption and water vapor resistance factors, as it has been shown in a few studies [48,49]. However, the obtained results are in excellent agreement with those for similar products existing on the market.

### 3.5. Thermal Stability and Flammability Properties of the Stable Products

**Figure 7:**
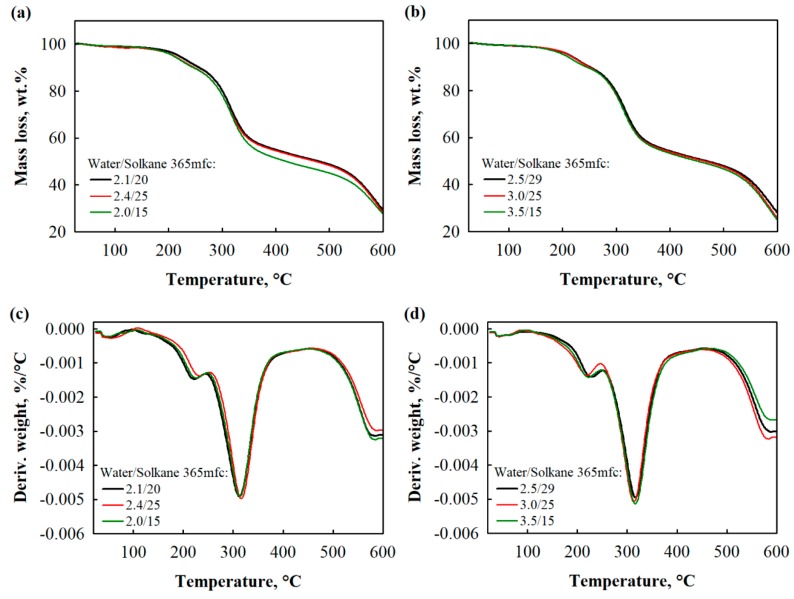
Thermogravimetry (TGA) and differential thermogravimetry (DTG) curves of polyurethane foams at an apparent density of (**a**,**c**) 40 kg/m^3^ and (**b**,**d**) 30 kg/m^3^.

Generally, thermal decomposition of polyurethane foams is a complex process due to extraction of many gaseous products [50]. Regarding the segmented structure of this type polymer degradation will be related to hard and soft segments. Well separated peaks on DTG curve (Figure 7c,d) provide with an additional information about phase separation of polyurethane foams with different water/Solkane 365 mfc ratios. It is seen that each polyurethane foam independently on blowing agents ratio decomposes in three steps and it confirms the segmented structure of the obtained foams.

At the first step at a temperature between 150 °C and 320 °C weight loss is attributed to dissociation of urethane bonds, while during the second step at a temperature between 320 °C and 420 °C soft is imputed to the degradation of soft polyol segments [51]. The third stage is assigned to the degradation of the fragments obtained during second stage degradation and it appears at the temperature of 480 °C. Additionally, char yield at 600 °C, peaks at corresponding stages and weight losses at 5 wt.% and 50 wt.% are presented in Table 7.

It is found that T_5 wt.%_, T_50 wt.%_ and T_max_ in all stages decrease as water content in blowing agents system increases from 2.1 to 3.5 pbw. The possible reason for this is related to crosslink-density and structure of polyurethane foams with different water/Solkane 365 mfc ratios. Water, as a chemical blowing agent, reacts with isocyanate thus generating polyurea and polybiuret together with CO_2_. Therefore, higher water content requires higher amount of isocyanate this way more polyurea and polybiuret form. As indicated in [52], polyurea and polybiuret are more rigid than polyurethane, so they should shift T_5 wt.%_, T_50 wt.%_ and T_max_ towards higher temperature. However, increasing amount of water reduces the crosslink density of polyurethane foams thus slightly reducing corresponding thermal degradation temperatures.

**Figure 8:**
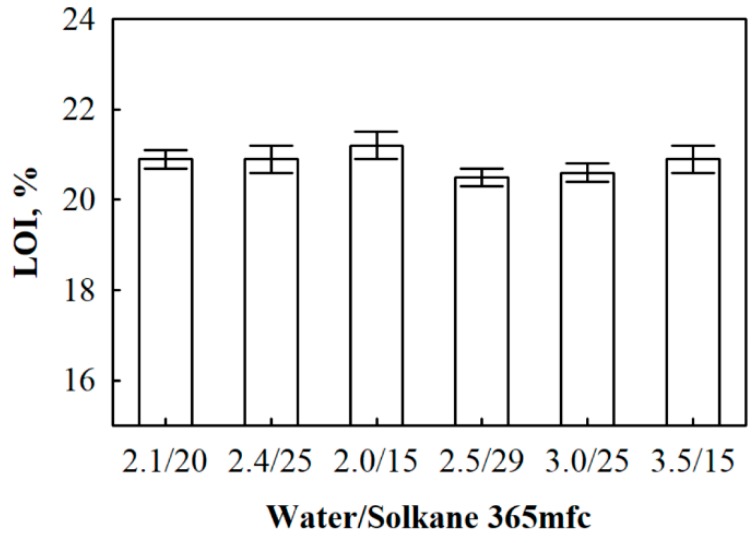
Limited oxygen index (LOI) test results for polyurethane foam with different water/Solkane 365 mfc ratios.

In order to determine the impact of water/Solkane 365 mfc ration on flammability, LOI tests ared conducted and obtained results are presented in Figure 8.

Obviously, polyurethane foam without flame retardants is flammable and its LOI value may reach only 19% [53]. The incorporation of flame retardant such as provided in Section 2.3 improves flammability properties and the LOI value of the obtained polyurethane foams irrespective of water/Solkane 365 mfc ratio varies from 20.6% to 21.2%. The difference is not high and varies within the margin of error for 40 and 30 kg/m^3^ density products. The obtained results show that the obtained foams are classified as slow burning materials.

## 4. Conclusions

The higher amounts of conventional blowing agent (15, 25, and 29 pbw) and water (2.5, 3.0, and 3.5 pbw) increase the expansion efficiency of the foam, resulting in product structures with ~2 times larger cells and products with an apparent density of ~30 kg/m^3^.

Irrespective of apparent density, the thermal conductivity before aging varies from 0.0196 to 0.0204 W/(m·K), while the value of thermal conductivity after aging ranges from 0.0256 to 0.0263 W/(m·K). The obtained values of the thermal conductivity are ensured by low diffusion of the blowing agent system. The replacement of 70% to 80% of difunctional polyols with multifunctional polyols results in dimensionally stable products at higher temperature and humidity conditions, showing that the resulting products exhibit dimensional changes no greater than 0.5% and meet the requirements of EN 14315-1 (≤ 10% in length and width and ≤ 15% in thickness) and EN 13165 (≤ 5% in length and width and ≤ 10% in thickness).

The moisture properties, short-term water absorption and the water vapor resistance factor, for the product varies from 0.22 to 0.35 kg/m^2^ and 19 to 50, respectively, in the density range of 30 to 40 kg/m^3^. The lower values of water absorption and the water vapor resistance factor are determined by adding 10% more of the multifunctional polyol. Therefore, the percent content of open cells decreases, thereby providing a barrier for water and water vapor penetration.

Thermal stability and flammability tests showed that the obtained polyurethane foams with different ratios off water/Solkane 365 mfc are characterized by almost the same properties. However, a slight increase in water reduces crosslink density of the foams thus shifting backwards the thermal degradation temperature. Additionally, all the obtained polyurethane foams can be considered as slow burning materials with an average LOI value varying from 20.6% to 21.2%.

## Figures and Tables

**Table 1:** Theoretically correct polyurethane foam compositions for two types of densities.

Material	Required Density, kg/m^3^	
	30	40
NEOPOLYOL 240	102.5	104
Glycerol	5.5	10.5
PETOL PZ 400-4G	36.5	34.5
Water	6.6	2.2
Solkane 365 mfc	22	25
Struksilon 8007	2	2
Polycat 9	4.2	4.2
Roflam P	44	37.5

**Table 2:** Basic chemical characteristics of polyols used in the production of rigid polyurethane foams.

Parameter	Value	
	NEOPOLYOL 240	PETOL PZ 400-4G
Hydroxyl value, mg KOH/g	240	425
Functionality, d. m. *	2.1	4.3
Density, g/cm^3^	1.23	1.10
Dynamic viscosity at 25 °C temperature, mPa·s	5250	7750
Acid value, mg KOH/g	<1	<1
Water content, %	≤0.1	≤0.1

* Dimensionless.

**Table 3:** Compositions of rigid polyurethane foams with different apparent densities.

Material	40 kg/m^3^ Water/Solkane 365 mfc				30 kg/m^3^ Water/Solkane 365 mfc			
	Control 2.2/25	2.1/20	2.4/25	2.0/15	Control 6.6/22	2.5/29	3.0/25	3.5/18
	Amount, pbw							
Neopolyol 240	104	30	30	30	102.5	20	20	20
Glycerol	10.5	-	-	-	5.5	-	-	-
Petol 400-4G	34.5	70	70	70	36.5	80	80	80
Water ^a^	2.2	2.1	2.4	2	6.6	2.5	3	3.5
Solkane 365 mfc	25	20	25	15	22	29	25	18
Struksilon 8007	2	2	2	2	2	2	2	2
Polycat 9	4.2	4.2	4.2	4.2	4.2	4.2	4.2	4.2
Roflam P	37.5	23	23	23	44	23	23	23
Isocyanate index ^b^	100							

^a^ Different amounts of water were estimated in the isocyanate content calculations; ^b^ The isocyanate index is the ratio of the equivalent to the theoretical isocyanate content multiplied by 100.

**Table 4:** Chemical characteristics of the polyol systems.

Blowing Agent Ratio, Water/Solkane 365 mfc	Parameter	
	Calculated Hydroxyl Value of Polyols Systems, mg KOH/g	Calculated Functionality of Polyols Systems
**40 kg/m^3^**		
Control 2.2/25	394	2.68
2.1/20	370	3.64
2.4/25		
2.0/15		
**30 kg/m^3^**		
Control 6.6/22	347	2.69
2.5/29	388	3.86
3.0/25		
3.5/15		

**Table 5:** Characteristic foaming times and temperatures.

Blowing Agent Ratio, Water/Solkane 365 mfc	Cream Time, s	String-gel Time, s	Tack-Free Time, s	The Highest Reaction Temperature, °C
**40 kg/m^3^**				
Control 2.2/25	5 ± 2	15 ± 1	44 ± 1	108 ± 3
2.1/20	3 ± 1	13 ± 2	44 ± 2	110 ± 2
2.4/25	3 ± 1	13 ± 1	42 ± 1	114 ± 2
2.0/15	3 ± 1	13 ± 1	44 ± 2	115 ± 3
**30 kg/m^3^**				
Control 6.6/22	4 ± 1	14 ± 1	43 ± 1	111 ± 2
2.5/29	3 ± 1	13 ± 1	43 ± 1	112 ± 2
3.0/25	3 ± 1	13 ± 1	41 ± 1	116 ± 3
3.5/15	3 ± 1	13 ± 1	41 ± 2	115 ± 2

**Table 6:** Average values of thermal conductivities and structural parameters of polyurethane foam.

Blowing Agents Ratio, Water/Solkane 365 mfc	Polyols Ratio, Neopolyol 240/Petol PZ 400-4G	Average Thermal Conductivity before Ageing, W/(m·K)	Average Thermal Conductivity after Ageing, W/(m·K)	Average Closed Cell Content, vol.%	Average Cell Size, mm
**40 kg/m^3^ apparent density**					
2.1/20	30/70	0.0203 ± 0.0001	0.0259 ± 0.0004	91.3 ± 1.4	0.373 ± 0.052
2.4/25	30/70	0.0203 ± 0.0002	0.0259 ± 0.0004	90.4 ± 1.6	0.382 ± 0.048
2.0/15	30/70	0.0204 ± 0.0001	0.0264 ± 0.0001	91.6 ± 1.3	0.394 ± 0.092
**30 kg/m^3^ apparent density**					
2.5/29	20/80	0.0201 ± 0.0001	0.0259 ± 0.0002	95.4 ± 2.0	0.784 ± 0.075
3.0/25	20/80	0.0203 ± 0.0001	0.0251 ± 0.0002	96.1 ± 1.8	0.788 ± 0.056
3.5/15	20/80	0.0198 ± 0.0003	0.0251 ± 0.0001	94.7 ± 1.4	0.782 ± 0.038

**Table 7:** Thermal degradation parameters of the stable polyurethane foams.

Water/Solkane 365 mfc	T_5 wt.%_, °C	T_50 wt.%_, °C	T_max_, °C			Char Yield at 600 °C, wt.%
			1st Stage	2nd Stage	3rd Stage	
**40 kg/m^3^**						
2.1/20	220	481	219	313	582	29.4
2.4/25	210	476	232	316	585	28.5
2.0/15	210	421	228	310	583	27.8
**30 kg/m^3^**						
2.5/29	214	468	225	318	589	28.2
3.0/25	213	464	222	312	585	26.0
3.5/15	205	452	224	320	592	25.1
